# Gene expression analysis of ovarian follicles and stromal cells in girls with Turner syndrome

**DOI:** 10.1093/molehr/gaae043

**Published:** 2024-12-03

**Authors:** Ron Peek, Sanne van der Coelen, Marie-Madeleine Dolmans

**Affiliations:** Department of Obstetrics and Gynaecology, Radboud University Medical Center, Nijmegen, The Netherlands; Department of Obstetrics and Gynaecology, Radboud University Medical Center, Nijmegen, The Netherlands; Gynaecology Research Unit, Institut de Recherche Expérimentale et Clinique, Université Catholique de Louvain, Brussels, Belgium; Department of Gynaecology, Cliniques Universitaires Saint-Luc, Brussels, Belgium

**Keywords:** Turner syndrome, aneuploidy, somatic cells, gene expression, mosaicism

## Abstract

In patients with mosaic Turner syndrome, the ovarian somatic cells (granulosa and stromal cells) display a high level of aneuploidy with a 45,X karyotype, which may affect gene expression in the ovary and contribute to their reduced fertility. The aim of the current research is to study the effect of aneuploidy of somatic ovarian cells on gene expression in ovarian cortex stromal cells and small ovarian follicles from mosaic (45,X/46,XX) Turner syndrome patients. To this end, ovarian cortical tissue was obtained by laparoscopic surgery from eight mosaic Turner syndrome patients (aged 5–19 years) and eight controls (aged 6–18 years). The tissue was fractionated to obtain purified follicles and stromal cells. Part of the purified fractions was used to determine the X chromosomal content of ovarian cells of Turner syndrome patients by interphase FISH, while the remaining part was used to compare the gene expression profile of these cells to controls. The results demonstrated that high level 45,X haploidy in cortical stromal cells of Turner syndrome patients had no effect on gene expression, gross morphology of the ovary, or histological appearance of the cortex compared to controls. Gene expression analysis of purified small follicles of Turner syndrome patients with mainly 45,X granulosa cells revealed aberrant expression of 11 genes. Of these, six were upregulated (*CD24*, *TLR1*, *EPHA2*, *PLXND1*, *ST6GALNAC5*, and *NOX4*) while five genes (*CRYAB*, *DLX1*, *PCYT2*, *TNFRSF8*, and *CA12*) were downregulated compared to follicles of controls. Interestingly, the overexpressed genes in these small follicles were all associated with more advanced stages of follicular development. The consequences of this abnormal gene expression in follicles for Turner syndrome patients remain to be investigated, but they are likely to affect fertility.

## Graphical Abstract

**Graphical Abstract:**
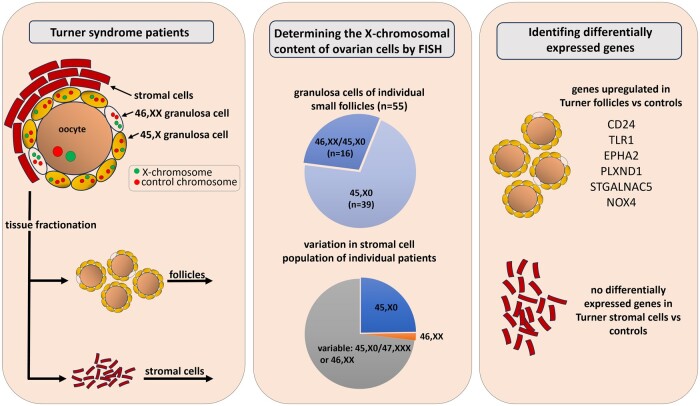
**Analysis of gene expression in 45,X/46,XX mosaic cells of ovarian follicles and stromal cells of young patients with Turner syndrome**.

## Introduction

Turner syndrome (TS) is one of the most common sex chromosomal disorders and affects approximately 1 in every 2500 girls. In TS, one of the X chromosomes is missing, partially missing, or altered, leading to a plethora of phenotypical and physiological aberrations, including thyroid disease, malformations of the heart and kidneys, hearing loss, and short stature (Ranke and Saenger, 2001; Gravholt, 2004).

Fertility is generally severely compromised due to germ cell apoptosis at the prophase of meiosis I and impaired folliculogenesis during the foetal stage of development, ultimately leading to a premature depletion of the ovarian reserve (Reynaud *et al.*, 2004; Lundgaard Riis *et al.*, 2021). A valid option to preserve fertility in TS patients could be cryopreservation of ovarian cortex tissue (OTC) at an early age at which the pool of primordial follicles is not yet exhausted (van der Coelen *et al.*, 2024). This is supported by the recent analysis of X chromosomal aneuploidy in the ovaries of young 45,X/46,XX mosaic TS patients that has shown that virtually all oocytes of the small (unilaminar primordial/primary) follicles have a normal 92,XXXX karyotype (Balen *et al.*, 2010; Peek *et al.*, 2019; Nadesapillai *et al.*, 2021; Schleedoorn *et al.*, 2022; Peek *et al.*, 2023). However, in contrast to the oocytes, the granulosa cells of these small follicles were found to be almost all aneuploid with a 45,X karyotype. The level of aneuploidy in the cortical stroma, in which these follicles are embedded, was generally high but varied considerably between TS patients (Peek *et al.*, 2019; Nadesapillai *et al.*, 2021; Schleedoorn *et al.*, 2022; Peek *et al.*, 2023). The high level of aneuploidy of the granulosa and cortical stromal cells might influence the capacity of small follicles to complete the complex process of folliculogenesis and hence the fertility potential of TS patients. Recent research by our group has shown that despite aneuploidy of the somatic cell compartment in the ovarian cortex, small follicles were capable of developing to morphologically normal secondary and antral follicles after xenografting into severe combined immunodeficient (SCID) mice (Peek *et al.*, 2023). Again, these observations substantiate that OTC might be used for fertility preservation in TS patients. However, normal folliculogenesis at the morphological level does not ensure that cytoplasmic and nuclear maturation of the oocyte, which are essential for successful fertilization, are achieved in the presence of aneuploid granulosa and stromal cells (Telfer and Anderson, 2023). The oocyte strongly depends on its enveloping granulosa cells for cytokines and growth factors that promote both repression and activation pathways (McLaughlin and McIver, 2009), but also on the stromal cell compartment that constitutes the follicular microenvironment, and from which the theca cells are recruited at more advanced stages of folliculogenesis. The absence of the second X chromosome in the somatic cells may therefore lead to aberrant gene expression that disturbs the complex bidirectional signalling between these somatic cells and the oocyte. This in turn could lead to altered gene expression in the follicle and disruption of oocyte maturation.

The goal of the current work is to analyse the gene expression of primordial/primary follicles and stromal cells of mosaic TS patients with a high level of aneuploidy in the ovary. To this end, we first determined the X chromosomal content of granulosa cells from individual follicles, and stromal cells from OTC of young mosaic 45,X/46,XX TS patients. Next, we analysed the mRNA expression pattern of purified follicles and stromal cells from OTC of these TS patients and age-matched controls. Profiling was performed by Targeted circularizing probe-based RNA next-generation sequencing (ciRNAseq) of 736 genes covering cellular processes such as proliferation, differentiation, adhesion, and cell–cell communication.

## Materials and methods

### Ethical approval

The use of human OTC from TS patients and controls was approved by the Dutch Central Committee on Research Involving Human Subjects (CCMO NL57738.000.16) and the Ethics Committee of the Cliniques Universitaires Saint-Luc, the Institutional Review Board of the Université Catholique de Louvain (institutional review board references 2012/23MAR/125 and 2020/11MAR/152). Written informed consent was obtained from all participants and/or their parents. Participants 16 years and older provided their own consent, participants aged 12 to 15 provided consent along with their parents, and for participants under 12, the parents provided consent.

### Participants

OTC fragments from eight patients with 45,X/46,XX mosaic TS (aged 5–19 years, mean age ± SD: 10.3 ± 4.7 years) and eight age-matched controls (aged 6–18 years, mean age ± SD: 11.5 ± 4.2 years) were used for this study. Tissue was obtained by laparoscopic ovariectomy and cryopreserved for fertility preservation purposes. The investigation is part of a nationwide trial ‘Preservation of Ovarian Cortex Tissue in Girls with Turner Syndrome’ (ClinicalTrials gov Identifier: NCT03381300) (Schleedoorn *et al.*, 2019). The characteristics of TS patients and controls are shown in Tables 1 and 2.

**Table 1.** Characteristics of the patients with mosaic Turner syndrome (TS) in lymphocytes.

Patient code	Age at OTC (years)	Karyotype lymphocytes [no. of cells]	FISH analysis		RNA isolation	
			No. of stromal cells	No. of follicles/granulosa cells	No. of stromal cells	No. of follicles
**TS1**	5	45,X[14]/46,XX[16]	100	11/227	>10 000	69
**TS2**	5	45,X[28]/46,XX[2]	100	5/91	>10 000	60
**TS3**	7	45,X[15]/46,XX[21]	100	11/219	>10 000	40
**TS4**	9	45,X[13]/46,XX[19]	100	3/89	>10 000	50
**TS5**	12	45,X[25]/46,XX[5]	100	7/149	>10 000	50
**TS6**	12	45,X[95]/46,XX[5]	100	10/220	>10 000	35
**TS7**	13	45,X[13]/46,XX[17]	100	7/144	>10 000	ND
**TS8**	19	45,X[26]/47,XXX[8]	100	1/12	>10 000	40

The number of stromal cells and follicles used for determining the X chromosomal content by FISH and isolation of RNA is indicated. Note that in TS8, an additional 47,XXX cell line was detected. ND, not done; OTC, ovarian tissue cryopreservation.

**Table 2.** Characteristics of control patients with oncological conditions (C1–C7) or gender dysphoria (C8).

Patient code	Age at OTC (years)	Diagnosis	Treatment before OTC	RNA isolation	
				No. of stromal cells	No. of follicles
**C1**	6	Ependymoma	Radiotherapy head	>10 000	ND
**C2**	7	Sarcoma	Chemotherapy (IVA and CEV) clavicular radiotherapy	>10 000	50
**C3**	8	Medulloblastoma	None	>10 000	35
**C4**	11	Sarcoma	None	>10 000	60
**C5**	13	Ewing sarcoma	None	>10 000	50
**C6**	14	NH lymphoma	None	>10 000	ND
**C7**	15	Ewing sarcoma	Euro Ewing 2008 protocol	>10 000	ND
**C8**	18	Gender dysphoria	Testosterone	>10 000	50

The number of stromal cells and follicles used for RNA isolation is indicated. Euro Ewing protocol consists of six cycles of vincristine, ifosfamide, doxorubicin and etoposide. ND, not done; OTC, ovarian tissue cryopreservation; NH, non-Hodgkin lymphoma.

### Slow freezing and thawing procedure

Ovarian tissue fragments from controls and TS patients were cryopreserved using a slow freezing/thawing protocol with dimethyl sulphoxide as cryoprotectant (Bastings *et al.*, 2014). One tissue fragment between 30 and 100 mg for TS patients and approximately 80 mg for controls was thawed and analysed for X chromosomal content and mRNA expression of ovarian cells.

### FISH analysis of ovarian cells

The thawed ovarian cortical tissue was subjected to enzymatic digestion to separate stromal cells and individual small follicles as detailed in Peek *et al.* (2019). The additional treatment of purified follicles with trypsin, as previously used to separate granulosa cells and oocytes more efficiently, was omitted as this interfered with subsequent RNA isolation (data not shown). The degree of aneuploidy was analysed by determining the X chromosomal content of stromal cells (*n* = 100 per TS patient) and granulosa cells (*n* = 12–227 per TS patient) from individual follicles (*n* = 1–11 per TS patient) by FISH using centromeric probes for the X chromosome (green) and chromosome 18 (red) for control purposes, as described before (Peek *et al.*, 2019).

### RNA isolation and reverse transcription

Part of the stromal cell suspension (>10 000 cells) and purified follicles (35–69 for TS patients; 35–60 for controls) was used for mRNA expression analysis. Total RNA was isolated using Tri-RNA reagent according to the manufacturer’s protocol (Favorgen, Ping-Tung, Taiwan). For Turner patient TS7 and control patients C1, C6, and C7, the number of purified follicles was too low for sufficient RNA extraction. RNA was reverse transcribed by Superscript II (Invitrogen, Carlsbad, CA, USA) using random hexamers (Promega, Madison, WI, USA). Integrity of the cDNA was verified by a 35-cycle intron-spanning PCR of *GAPDH* using forward primer 5′-TGGGTGTGAACCATGAGAAG-3′ and reverse primer 5′-AGTTGTCATGGATGACCTTGG-3′, this indicated that the cDNA did not contain detectable chromosomal DNA.

### Targeted gene expression analysis

cDNA generated from RNA extracted from purified follicles and stromal cells was used to analyse gene expression using ciRNAseq (Predica Diagnostics, Nijmegen, The Netherlands). The protocol for ciRNAseq was described before (de Bitter *et al.*, 2017). In short, single molecule Molecular Inversion Probes (smMIPs) to detect gene transcripts with known involvement in follicular development, were designed and added to a previously described probe set (Andralojc *et al.*, 2022). Transcript sequences were retrieved from hg38 (https://www.ncbi.nlm.nih.gov/assembly/GCF_000001405.26/). cDNA from follicles and stromal cells was captured overnight with the final set of smMIPs to identify and quantitatively measure expression levels of a total of 736 gene transcripts, involved in general cell metabolism, tyrosine kinase signalling, cell proliferation, tumour suppression, genes involved in immunity and genes know to be specifically expressed in oocytes and follicular granulosa cells. Illumina-barcoded CiRNAseq libraries were pooled and sequenced on a NOVASEQ6000 SP1 flow cell (Illumina, Eindhoven, The Netherlands). Decomplexed FASTQ data were processed to normalized gene expression levels by ciRNAlyzer software (Predica Diagnostics). Follicles isolated from tissue of Turner patient TS7 and control patients C1, C6, and C7 did not yield sufficient RNA for ciRNAseq analysis.

### Statistical analysis

Samples with sequencing depth (expressed as a number of circularized smMIPs during capture (de Bitter *et al.*, 2017)) below threshold were omitted from the analysis. Gene transcript levels were normalized and expressed as fragment per million (FPM). After log transformation, a two-tailed Student’s *t*-test was performed to identify differential gene expression between TS follicles versus TS stroma; control follicles versus control stroma; TS stroma versus control stroma, and TS follicles versus control follicles. In addition, differential gene expression between groups based on the level of aneuploidy was compared: TS stroma (5–9 years) versus TS stroma (12–19 years). Fold change was calculated and considered significant at *P* < 0.05.

## Results

### Selection of TS patients and controls

**Figure 1.**
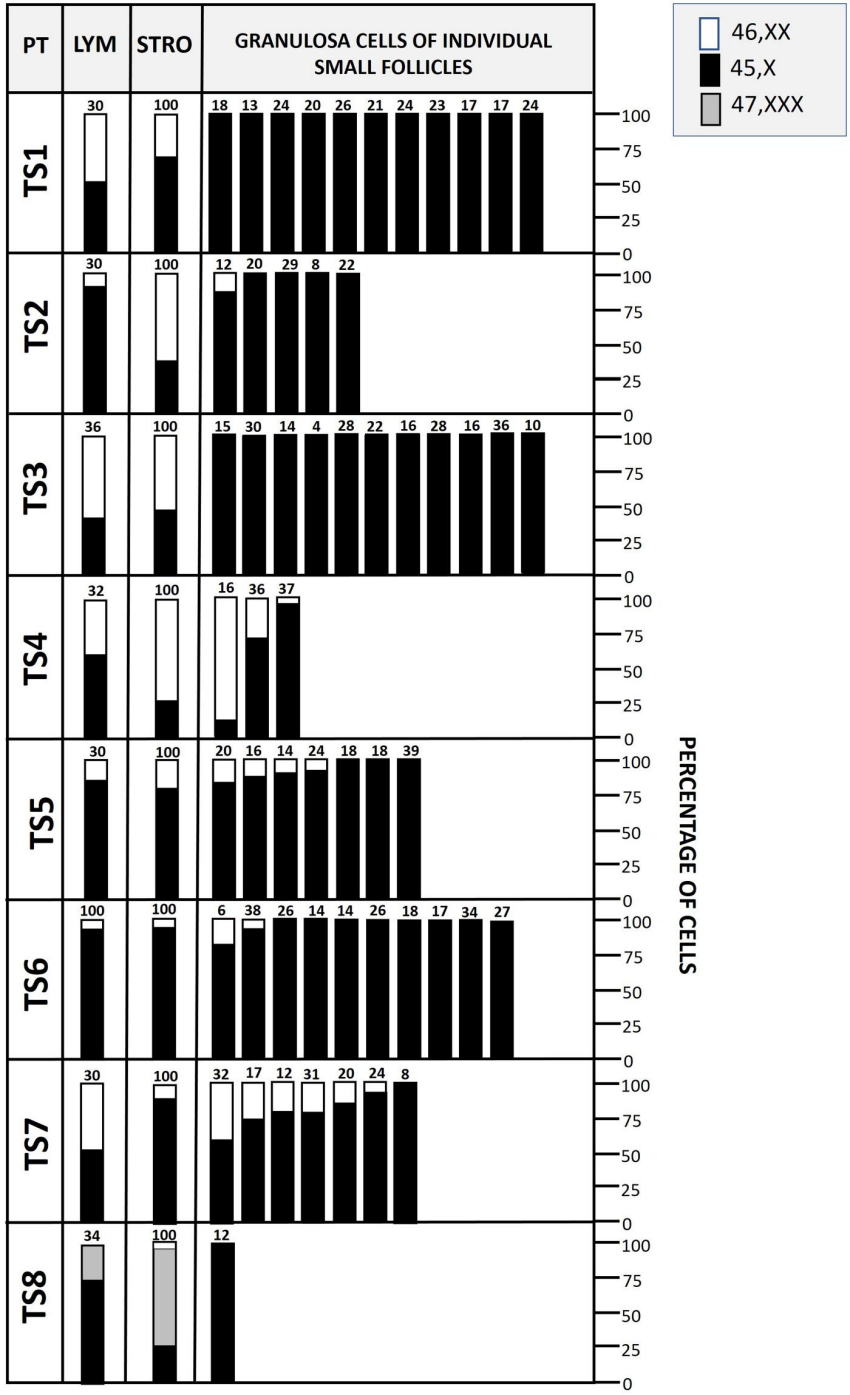
**X chromosomal mosaicism in lymphocytes (LYM, *n* ≥ 30 cells), ovarian stromal cells (STRO, *n* = 100 cells), and granulosa cells of small follicles from Turner syndrome patients (TS)**. X chromosomal content of cells was analysed by FISH with an X chromosomal-specific probe. The percentage of cells with a particular karyotype is indicated. In the rightmost panels, the percentage of granulosa cells with a particular karyotype is shown for individual small follicles, each bar represents a single small follicle. The number above each bar indicates the number of cells that were analysed. Patients TS1-7 were 45,X/46,XX mosaic while in patient TS8, an additional 47,XXX cell line was detected.

From the 97 TS patients that were included in our nationwide trial (Nadesapillai *et al.*, 2023), eight patients aged 5–19 years (TS1–8) with a non-structural mosaic karyotype 45,X/46,XX (/47,XXX) in lymphocytes were selected for the current study, based on the presence of ovarian follicles in the ovarian cortex (Table 1). In the lymphocytes of seven of these mosaic TS patients (TS1–7) 45,X and 46,XX cell lines were found, while TS8 displayed an additional 47,XXX cell line. The percentage of aneuploid cells varied considerably between TS patients. For example, in TS6, nearly all lymphocytes had a 45,X karyotype, while in TS3, the majority of lymphocytes displayed a normal 46,XX karyotype (Fig. 1). Control OTC fragments were obtained from age-matched individuals aged 6–18 years undergoing oophorectomy for fertility preservation purposes, before fertility threatening cancer treatment (C1 and C3–6), after chemo-/radiotherapy (C2 and C7), or as part of gender-affirming surgery (C8) (Table 2).

### Fractionation of ovarian cortex tissue for FISH and gene expression analysis

**Figure 2.**
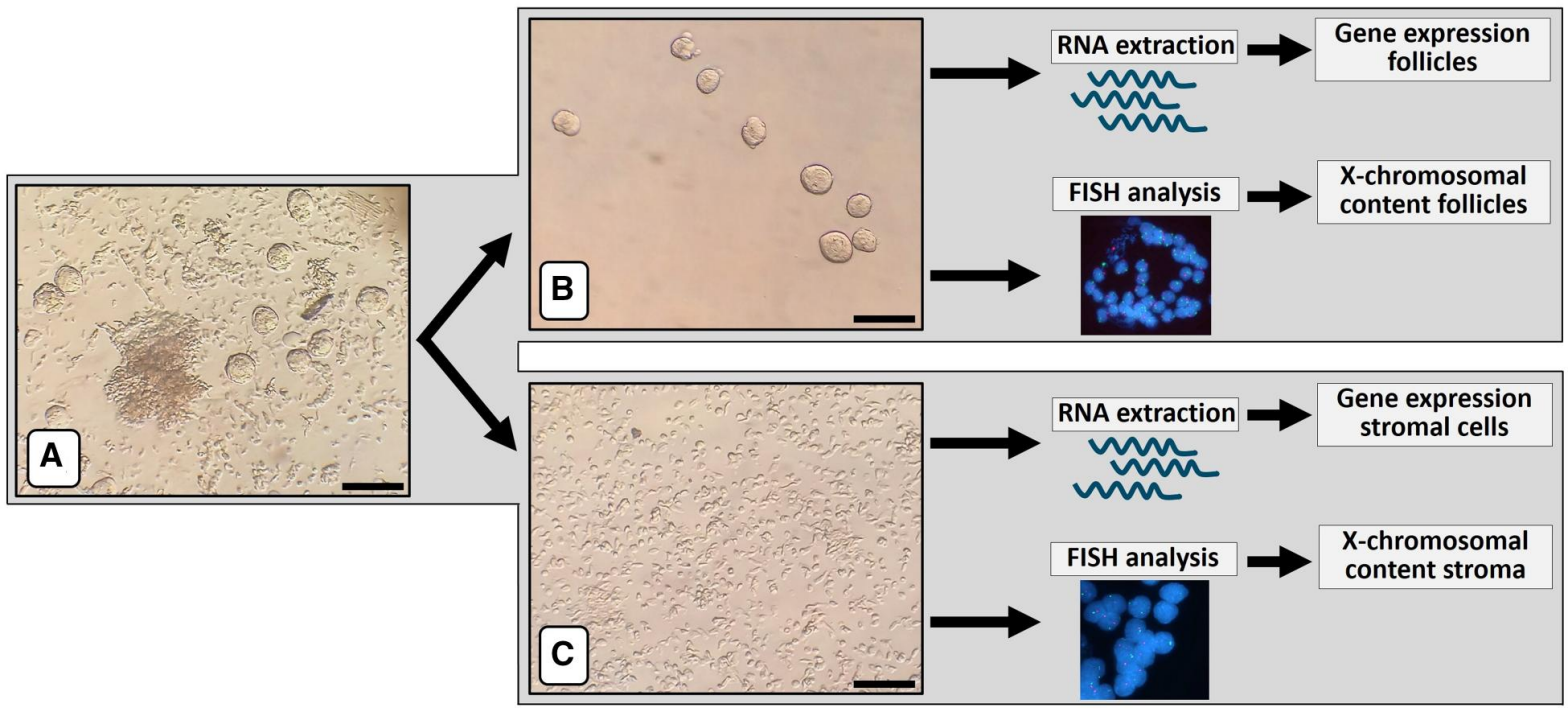
**Flow scheme illustrating the separation of ovarian stromal cells and follicles from Turner syndrome patients and controls.** An ovarian cortex fragment was subjected to enzymatic digestion (**A**). Small follicles were manually picked up from the cell suspension and further purified by a second round of selection (**B**), leaving a stromal cell suspension devoid of follicles (**C**). Both follicle and stromal cell suspensions were then divided, part being used for RNA extraction and subsequent gene expression analysis, while the other part was used to determine the X chromosomal content of stromal cells and granulosa cells from individual follicles by interphase FISH with chromosome X (green) and chromosome 18 (red)-specific probes. Bars represent 100 µm. Original magnification of FISH signals was ×630.

**Figure 3.**
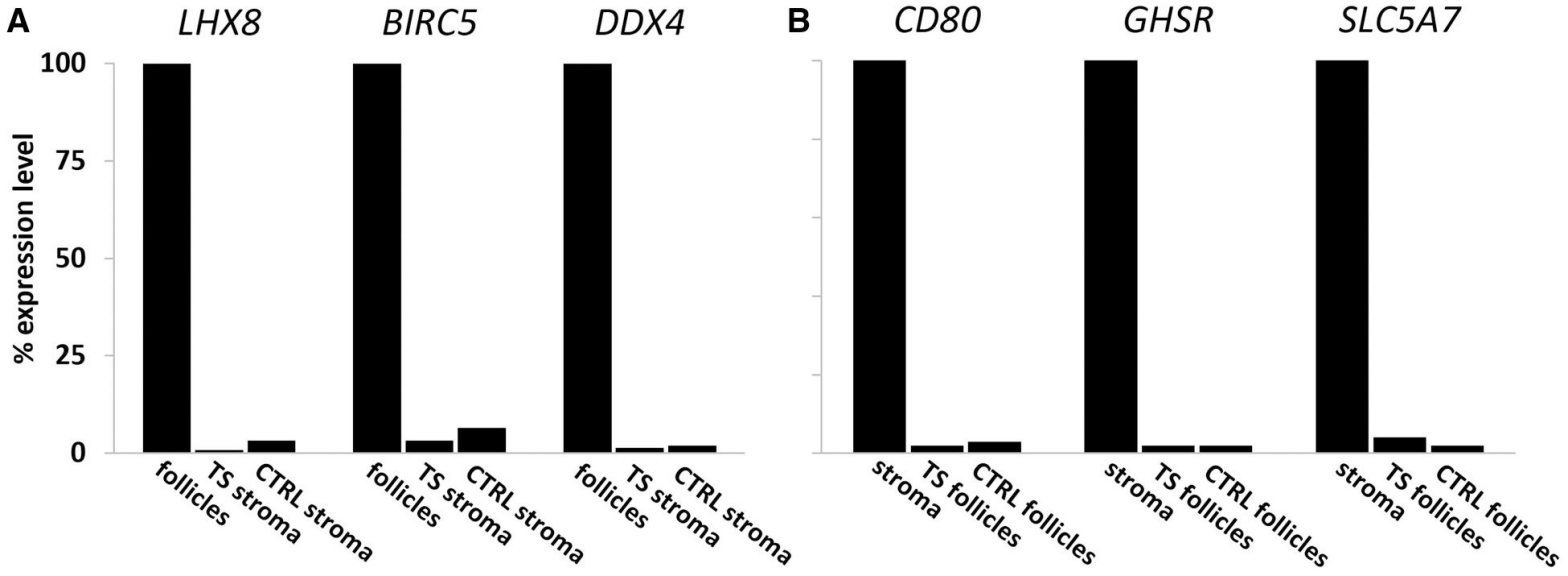
**Differential gene expression in purified ovarian follicles and stromal cells demonstrates efficient separation of ovarian cortex constituents.** RNA from small follicles and stromal cells was analysed by Targeted circular probe-based RNA next-generation sequencing (ciRNAseq). Transcripts of a large number of genes were differentially expressed. LIM homeobox 8 (*LHX8*), Baculoviral IAP repeat-containing protein 5 (*BIRC5*), and DEAD-Box Helicase 4 (*DDX4*) could be readily detected in follicles of both Turner syndrome (TS) patients and controls (CTRL) but were virtually absent in the corresponding stromal cells (**A**). Conversely, genes such as *CD80*, growth hormone secretagogue receptor (*GHSR*), and solute carrier family 5 member 7 (*SLC5A7*) were expressed at a much lower level in follicles compared to stromal cells (**B**). Gene expression was set at 100% in follicles (**A**), or in stromal cells (**B**).

The gene expression pattern and level of X chromosomal aneuploidy in ovarian follicles and stromal cells were analysed separately. To this end, the cortex tissue was digested enzymatically to obtain purified follicles and stromal cells devoid of follicles (Fig. 2). Part of these purified fractions from both TS patients and controls were used for gene expression analysis. In addition, purified follicles and stromal cells from TS patients were also subjected to FISH analysis to determine the level of X chromosomal aneuploidy. Both FISH and gene expression analysis indicated that the separation of follicles and stromal cells was highly effective. For example, in cortex tissue from TS1, 30% of the stromal cells were 46,XX, while all of the 227 granulosa cells of 11 purified follicles were 45,X. This indicates that these purified follicles were not contaminated with stromal cells (Fig. 1). Gene expression analysis also indicated effective separation of stromal cells and follicles. Several gene products known to be expressed in granulosa cells and/or oocytes such as *LHX8*, *BIRC5* (Survivin), and *DDX4* were indeed present in follicles of both TS patients and controls, but hardly detectable in stromal cells of the same tissue fragment. In contrast, transcripts of genes such as *CD80*, *GHSR*, and *SLC5A7* were readily detectable in stromal cells but only at very low levels in the follicular fractions (Fig. 3). These results indicate that enzymatic dissociation of the OTC, and subsequent separation of stromal cells and follicles results in purified cellular fractions that can be reliably used for further analysis of X chromosomal content and differential gene expression.

### Determining the X chromosomal content of ovarian follicles and cortex stromal cells in TS patients

FISH analysis of the number of X chromosomes in stromal cells revealed that the level of aneuploidy varied considerably between TS patients. For example, in TS2, TS3, and TS4, the majority of stromal cells were 46,XX, while in TS5, TS6, TS7, and TS8, almost all of these cells were aneuploid. The X chromosomal content of granulosa cells from individual small follicles was more consistent. In TS1 and TS3, none of the 22 small follicles that could be karyotyped contained a euploid granulosa cell (Fig. 1). The ratio between 45,X and 46,XX granulosa cells was different not only between patients but also between follicles of the same patient (e.g. TS7). Although some follicles contained several euploid granulosa cells, the vast majority was found to be 45,X.

### Analysis of gene expression in ovarian cortex stromal cells

**Figure 4.**
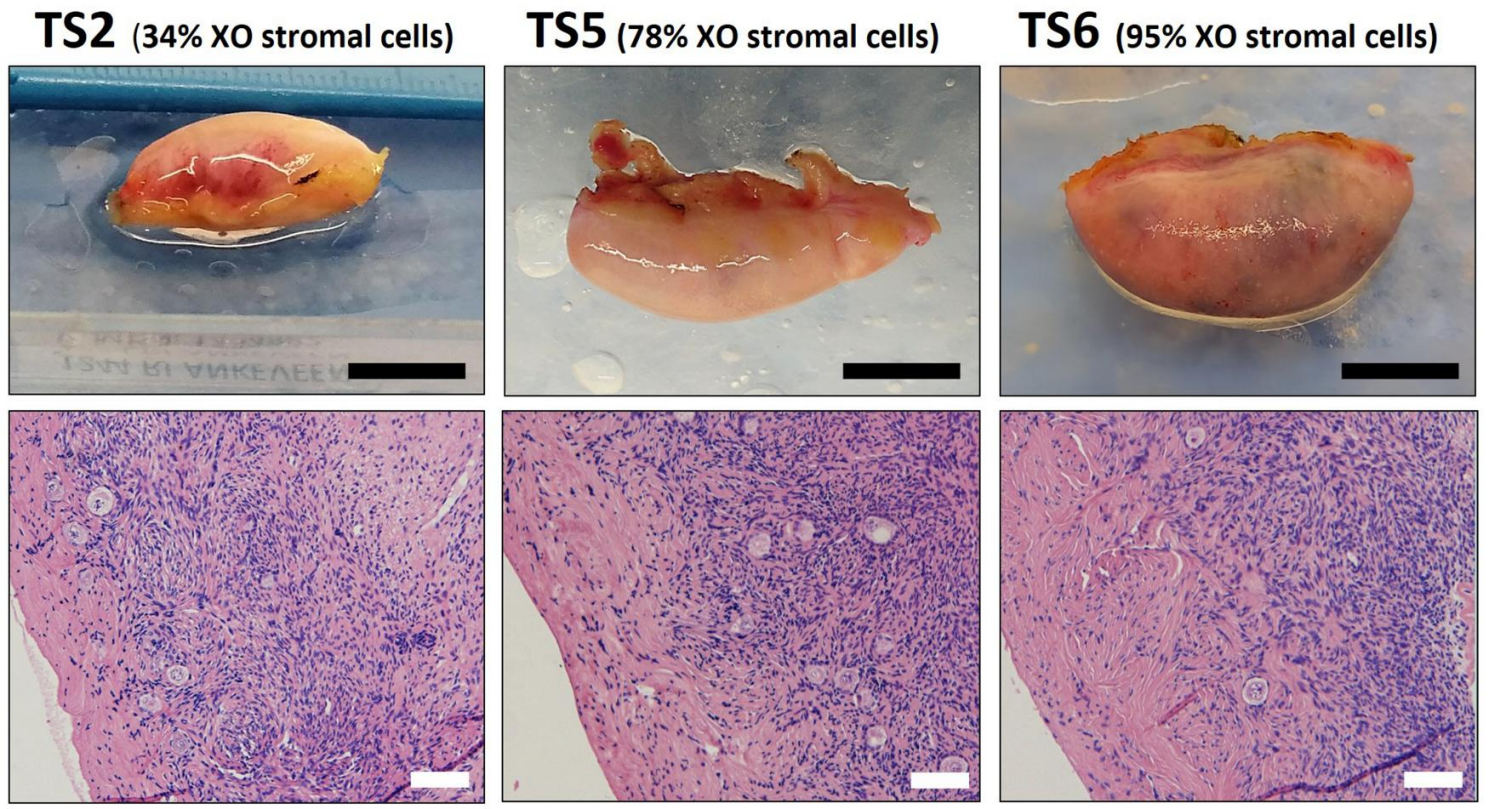
**Macroscopic and histological analysis of ovaries from Turner syndrome patients**. Ovaries containing 34% up to 95% of aneuploid cortex stromal cells do not show any gross morphological abnormalities. Histology of the ovarian cortex indicates normal levels of extra-cellular matrix and tissue cellularity. Bars indicate 1 cm (top panels) or 100 µm (bottom panels). TS, Turner syndrome.

Stromal cells from TS patients (*n* = 8; Table 1) and controls (*n* = 8; Table 2) were analysed for the expression of 736 individual genes by ciRNAseq. Despite the high level of aneuploidy in the stromal cells of most TS patients (Fig. 1), no statistically significant differences in gene expression between TS patients and controls were observed in these cells. Macroscopic images of ovaries from TS patients with different percentages of aneuploid stromal cells (TS2: 34%, TS5: 78%, or TS6: 95%) directly after oophorectomy indicated normal gross morphology. Further histological examination of the stromal cell compartment of the cortex of these ovaries also revealed no obvious differences between these tissues, despite the large difference in percentage of 45,X stromal cells (Fig. 4).

In view of the significant differences between TS patients in the level of aneuploidy in their stromal cells (Fig. 1), we compared the gene expression pattern of stromal cells with relatively low-level mosaicism (TS1, TS2, TS3, and TS4) with stromal cells displaying high-level mosaicism (TS5, TS6, TS7, and TS8). From these analyses several interesting gene products were identified that were significantly lower expressed (*P* < 0.05) in the stromal cells of TS patients with high-level mosaicism compared to TS patients with low-level mosaicism in their stromal cells, including DDX4, DHH, MYBL2, CSF1R, and ERBB2.

### Analysis of gene expression in ovarian follicles

The gene expression of purified follicles from TS patients and age-matched controls was analysed by ciRNAseq. This technique allows for the quantitative detections of mRNAs of interest with high sequencing depth and has been successfully used to analyse gene expression in various tissues (de Bitter *et al.*, 2017; Lenting *et al.*, 2019; van den Heuvel *et al.*, 2020; Andralojc *et al.*, 2022). A total of 11 genes displayed a statistically significant difference in expression between TS patient-derived follicles and follicles from controls (fold change >3; α = 0.05). The expression of six of these genes (*CD24*, Toll-like receptor 1 (*TLR1*), ephrin receptor A2 (*EPHA2*), plexin D1 (*PLXND1*), ST6 N-acetylgalactosaminide alpha-2,6-sialyltransferase 5 (*ST6GALNAC5*), and NADPH oxidase 4 (*NOX4*)) was significantly upregulated in TS follicles and five genes (crystallin alpha B (*CRYAB*), distal-less homeobox 1 (*DLX1*), phosphate cytidylyltransferase 2 (*PCYT2*), TNF receptor superfamily member 8 (*TNFRSF8*), and carbonic anhydrase 12 (*CA12*)) were downregulated compared to control follicles (Table 3).

**Table 3.** Differentially expressed genes in ovarian follicles from TS patients.

Differentially expressed genes	Function (in reproduction)	Expression in Turner follicles	Expression in control follicles	Fold change Turner/controls	*P*-value
***CD24***	Cell adhesion molecule (mediator of ovulation, expressed in granulosa cells of antral follicles)	106.2	0.0	–	0.021
***TLR1***	Pathogen recognition (poor ovarian response when elevated, TLRs in preovulatory follicles)	65.6	4.7	14.0	0.010
***EPHA2***	Tyrosine kinase (expression in granulosa corpus lutein cells)	114.7	27.5	4.2	0.030
***PLXND1***	Cell surface receptor (corpus lutein tropism)	121.3	29.4	4.1	0.048
***ST6GALNAC5***	Sialyltransferase (poor ovarian response)	107.2	32.6	3.3	0.023
***NOX4***	NADPH oxidase (granulosa cell proliferation)	369.0	115.8	3.2	0.001
***CRYAB***	Heat shock protein (no data available)	13.2	64.4	0.2	0.013
***DLX1***	Transcription factor (decrease from infancy to puberty)	4.4	24.3	0.2	0.032
***PCYT2***	Phosphate cytidyltransferase (no data available)	15.7	107.9	0.1	0.003
***TNFRSF8***	TNF receptor (no data available)	2.4	40.2	0.1	0.013
***CA12***	Carbonic anhydrase (no data available)	0.6	29.3	0.0	0.032

The RNA expression pattern of follicles from TS patients and controls was determined by ciRNAseq. Six genes were upregulated (*CD24*, *TLR1*, *EPHA2*, *PLXND1*, *ST6GALNAC5*, and *NOX4*) and five genes were downregulated (*CRYAB*, *DLX1*, *PCYT2*, *TNFRSF8*, and *CA12*) in small follicles of TS patients compared to controls (fold difference >3; *P* = 0.05). The general function of the corresponding gene products and their role in folliculogenesis and/or female reproduction is indicated in brackets. *TLR1*, Toll-like receptor; *EPH2*, ephrin receptor A2; *PLXND1*, plexin D1; *ST6GALNAC5ST6*, N-acetylgalactosaminide alpha-2,6-sialyltransferase; *NOX4*, NADPH oxidase 4; *CRYAB*, crystallin alpha B; *DLX1*, distal-less homeobox 1; *PCYT2*, phosphate cytidylyltransferase 2; *TNFRSF8*, TNF receptor superfamily member 8; *CA12*, carbonic anhydrase 12.

**Figure 5.**
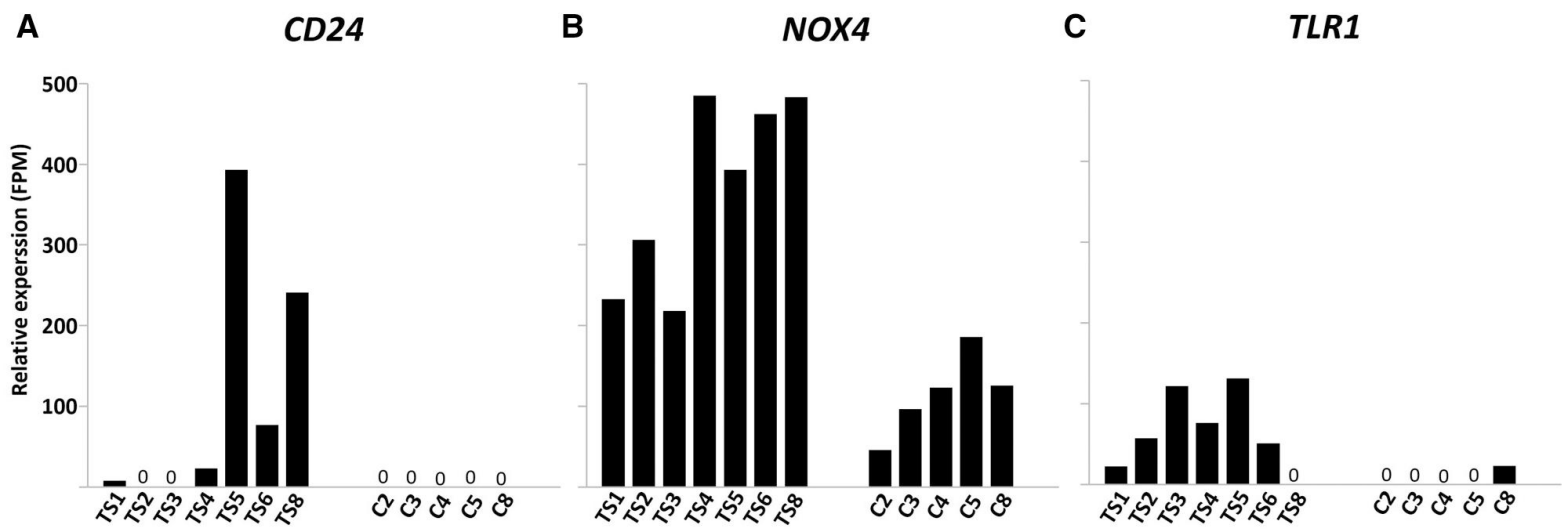
**Individual variation of differentially expressed genes.** The expression levels of genes showing statistically differential expression between small follicles of Turner syndrome patients (TS1–TS6 and TS8) and controls (C2–C5 and C8) indicated considerable individual variation. For example, *CD24* could not be detected in the follicles of control patients but was also undetectable in the follicles of two TS patients (**A**). Similar observations were made for the expression of Toll-like receptor 1 (*TLR1*) (**C**). Expression of NADPH oxidase 4 (*NOX4*) was upregulated for each TS patient compared to the controls, but also here expression levels varied more than 2-fold (**B**). FPM, fragment per million.

Considerable variation in expression level was observed among both TS patients and controls. For example, expression of CD24 could not be detected in follicles of controls but was also undetectable in two TS patients (Fig. 5A); transcripts of NOX4 were present in all follicle preparations but at a higher level in follicles from TS patients compared to controls (Fig. 5B), and expression of TLR1 was readily detectable in follicles of six out of seven TS patients but in only one of the control patients (Fig. 5C).

## Discussion

Preservation of fertility in women with TS by cryopreservation of either oocytes or OTC is currently being performed in many countries (Oktay *et al.*, 2016; Schleedoorn *et al.*, 2019; Nadesapillai *et al.*, 2023). Especially in mosaic 45,X/46,XX TS patients, the ovarian reserve might be sufficient to provide a realistic chance of storing enough gametes for establishing a pregnancy later in life (Donnez and Dolmans, 2023; Nadesapillai *et al.*, 2023). This is substantiated by recent findings showing that oocytes of small (primordial and primary) follicles in young women with mosaic TS have a normal 92,XXXX karyotype. Furthermore, small follicles of mosaic 45,X/46,XX TS patients have been shown to progress to morphologically normal secondary and antral follicles after xenografting to SCID mice (Peek *et al.*, 2023). However, control of primordial follicle activation and subsequent maturation during folliculogenesis depends on complex bidirectional signalling between the oocyte and the surrounding somatic cells (McLaughlin and McIver, 2009). Recent unanticipated findings have indicated that the granulosa cells of small follicles in mosaic TS are mostly all 45,X, while the cortical stromal cells contain a more variable level of aneuploidy (Peek *et al.*, 2019). Although the development of morphologically normal secondary/antral follicles in the xenograft experiments (Peek *et al.*, 2023) suggests that folliculogenesis is normal in mosaic TS patients despite this aneuploidy, the absence of a second X chromosome in the somatic cells may have adverse effects on cell–cell interaction, leading to aberrant gene expression in the oocyte. Recent observations have indicated that, although the second X chromosome (Xi) is largely transcriptionally silenced in normal 46,XX cells, genes that escape silencing on Xi modulate expression on the active X chromosome and up to 21% of the genes located on the autosomes, depending on the cell type analysed (San Roman *et al.*, 2023, 2024). The absence of the ‘silenced’ Xi chromosome in the 45,X ovarian somatic cells in TS could therefore have major implications for the close communication between the oocyte and the granulosa/stromal cells required for the proper initiation and continuation of folliculogenesis.

To investigate the effects of an absent Xi on gene expression in the somatic cells, purified small follicles and stromal cells from eight young patients with mosaic 45,X/46,XX TS and age-matched controls were analysed by ciRNAseq. This technique allowed us accurately to quantify differential expression of several hundreds of genes between these two groups. Although ciRNAseq is not designed to analyse the complete gene expression profile, it is less expensive and more suitable to quantify relative gene expression than whole-transcriptome or RNA sequencing (Arts *et al.*, 2017). Follicles were considered as a functional unit and not further fractionated into granulosa cells and oocytes, to prevent disruption of the intimate connection between the oocyte and the granulosa cells that may skew gene expression. In addition to ciRNAseq analysis, we determined the level of X chromosomal aneuploidy in granulosa cells of purified small follicles and ovarian cortex stromal cells in the TS patients. In line with previous results, the granulosa cells were almost exclusively 45,X, while the percentage of aneuploid cells in stromal cells varied considerably between TS patients. The X chromosomal content of the oocytes was not analysed in this study as we and others have previously shown that over 95% of oocytes in mosaic TS have a normal 92,XXXX karyotype (Balen *et al.*, 2010; Peek *et al.*, 2019; Nadesapillai *et al.*, 2021; Schleedoorn *et al.*, 2022; Peek *et al.*, 2023). Mechanisms that check homologous synapsis in the oocyte during prophase of meiosis I are probably the reason why oocytes that lack X chromosomes are largely eliminated from the pool of small follicles (Reynaud *et al.*, 2004; Burgoyne *et al.*, 2009). Similar to women with mosaic TS, the majority of germ cells in a 45,X/46,XY mosaic man was also found to have a normal chromosomal constitution (Ren *et al.*, 2015). This suggests that during both female and male gametogenesis, similar mechanisms ensure a normal karyotype in gametes.

Comparing the gene expression profile of ovarian cortex stromal cells of the eight TS patients with stromal cells of controls did not show significant differential expression of gene products. This was substantiated by gross morphology of the intact ovary and histology of the ovarian cortex. These findings may explain why some tissues are not affected, or less affected, in patients with TS, although the level of mosaicism is also likely to contribute to the variation in severity of phenotypical features and symptoms associated with TS. The level of aneuploidy in the stromal cells varied considerably between TS patients. Comparing the expression profile of TS patients with a relatively low frequency of aneuploid stromal cells (TS1–TS4) to that of patients with a high proportion of aneuploid stromal cells (TS5–TS8), revealed differential expression of several interesting genes encoding proteins involved in female reproduction. These include the germline-specific protein DDX4 that is commonly used as a marker for germ cells but is also expressed in perivascular cells (Wagner *et al.*, 2020); DHH, involved in gonadal genesis (Neocleous *et al.*, 2019); MYBL2, implicated in autophagy suppression and female fecundity (Yuan *et al.*, 2015); BUB1, a spindle assembly checkpoint protein for spindle assembly, which at low levels leads to aneuploidy (Baker *et al.*, 2004); CSF1R, overexpressed in poor-quality embryos (Yu *et al.*, 2023) and ERBB2, which initiates primordial follicle growth (Li-Ping *et al.*, 2010). Although the TS patients with low-level mosaicism in the stromal cells were all younger (5–9 years) than those with high-level mosaicism (12–19 years), it is unlikely that the differences in gene expression are related to age, as none of these genes showed differential expression related to age in the controls (results not shown).

Gene expression analysis of small follicles of mosaic TS patients indicated upregulation of six genes (*CD24*, *TLR1*, *EPHA2*, *PLXND1*, *ST6GALNAC5*, and *NOX4*) and downregulation of five genes (*CRYAB*, *DLX1*, *PCYT2*, *TNFRSF8*, and *CA12*), compared to controls. For the downregulated genes, no obvious link with folliculogenesis could be identified and their influence on fertility remains obscure for now. The relation to folliculogenesis and fertility of the genes that are upregulated in TS follicles is more obvious. Signal transducer CD24 is a marker of granulosa cell subpopulations and involved in ovulation. The expression of the CD24 protein is negligible in small follicles but increased in granulosa cells of antral follicles and corpus luteum in women with normal ovarian function (Dong *et al.*, 2019). This is in line with our findings that *CD24* transcripts were undetectable in control follicles but overexpressed in small follicles of TS patients. Also, *TLR1* expression was significantly upregulated in follicles of TS patients and only detectable at low level in one of the controls. Induction of Toll-like receptor gene expression has been described for granulosa cells of preovulatory follicles after LH induction (Hernandez-Gonzalez *et al.*, 2006; Shimada *et al.*, 2006). Furthermore, expression of *TLR1* was higher in patients with poor ovarian response (Taghavi *et al.*, 2014). Expression of *EPHA2* gene has been located in human granulosa lutein cells that arise after ovulation (Xu *et al.*, 2006) and was overexpressed in small follicles of TS patients in our study. There are no published data on the role of PLXND1 in human folliculogenesis but in buffalo, this protein plays a critical role in corpus luteum development (Jerome *et al.*, 2020). A similar role for PLXND1 could be envisioned in humans. The *ST6GALNAC5* gene codes for a Golgi type II transmembrane glycosyltransferase and showed a 3-fold overexpression in TS follicles. Increased expression of *ST6GALNAC5* in granulosa cells after ovarian stimulation has been reported (Liu *et al.*, 2022). The overexpression of the *NOX4* gene in the TS follicles could interfere with the production of H_2_O_2_ in granulosa cells. NOX-4-derived H_2_O_2_ has been shown to promote granulosa cell proliferation (Buck *et al.*, 2019). The overexpression of *NOX4* might be related to the rapid and preferential expansion of 46,XX granulosa cells to compensate for the impaired growing capacity of 45,X granulosa cells in follicles of TS patients (Nielsen and Krag-Olsen, 1980; Peek *et al.*, 2023). These observations indicate that the genes identified in this study that are upregulated in small follicles of TS patients are associated with more advanced stages of folliculogenesis, or even with functions of terminally differentiated granulosa cells in the corpus luteum after ovulation.

The inter-individual variation in gene expression we observed between TS patients, but also between controls, was sometimes considerable. For example, expression of the cell adhesion molecule CD24 was undetectable in the controls but highly variable in TS patients, while the magnitude of expression of NOX4 differed up to a factor of 4 in the controls. Possibly, expression levels of genes such as CD24 and NOX4 in follicles of TS patients are subject to age, but in this article, the number of TS patients and controls is too low for reliable statistical analysis of age-related gene expression differences in follicles. Gene expression differences between individuals have been described before and are generally limited to 2.5-fold (Eady *et al.*, 2005; Storey *et al.*, 2007). In addition to this natural gene expression variation, the differences observed in ovarian follicles could be related to heterogeneity in the pool of small follicles (Mamsen *et al.*, 2019; Peek *et al.*, 2023) and epigenetic mechanisms such as DNA methylation-dependent gene enhancing or silencing effects that have been described in cells of TS patients (Trolle *et al.*, 2016; Nielsen *et al.*, 2020). Furthermore, gene expression in the ovarian cells of some of the control patients might have been influenced by their treatment before ovariectomy.

Future research on gene expression in ovarian cells of TS patients should also focus on age-dependent differences and expression differences related to the level of aneuploidy in a larger number of patients. The recruitment of a sufficient number of TS patients for such a study, however, will be very challenging.

Taken together, our study provides evidence for aberrant gene expression in small follicles of young mosaic TS patients. The genes that were overexpressed all point to a more advanced follicular phenotype. The effect of this deviant gene expression on the ovarian reserve, the validity of the use of fertility preservation in TS, and ultimately on the fertility potential of TS patients remains to be investigated.

## Data Availability

The data underlying this article will be shared on request to the corresponding author. Predica Diagnostics will share the processed ciRNAseq data upon reasonable request in the context of collaboration programmes and under a Confidential Disclosure Agreement.
